# Impact of forestry on environment and human health: an evidence-based investigation

**DOI:** 10.3389/fpubh.2023.1260519

**Published:** 2023-09-01

**Authors:** Abdullah Addas

**Affiliations:** ^1^Department of Civil Engineering, College of Engineering, Prince Sattam Bin Abdulaziz University, Al-Kharj, Saudi Arabia; ^2^Landscape Architecture Department, Faculty of Architecture and Planning, King Abdulaziz University, Jeddah, Saudi Arabia

**Keywords:** the health impact of the forest, forest and health, Green Care Forest, strategic measures, the recreational function of the forest, sustainability

## Abstract

There is an increasing interest in the health effects of the forest. Without active participation in forestry, conflicts between the various stakeholders are foreseeable. The impact of forests on human life is unforgettable, and everyone gets enormous benefits from trees and greenery. COVID-19 has caused many changes in human behavior, which needs much attention. Environmental change's impact is considered a better solution and influences human behavior. Scientists around the globe are conducting research experiments on trees and the effect of forestry on human health, which is increasing in terms of social, ecological, and economic services. Trees provide full support to enhance the quality of life and minimize air pollution. Forests must be noticed to get benefits (e.g., carbon storage, fruits, human health considerations, economic benefits, and biodiversity). This research aims to explain the area of forest and health from the perspective of Saudi Arabian forestry and develop strategic measures for the proactive design of this topic. The research entailed expert interviews with forestry representatives and a quantitative survey of medical students. Our findings show that implementing strategic measures, such as establishing a forest and health, improves health and eliminates air pollution. It shows many other establishment and planning strategies, such as the use of professional visitor monitoring, the development of product innovations, the use of digitalization, and the development of integrated forest management.

## 1. Introduction

Since around 2011, Arabian forestry has been dealing with the health effects of the forest. There are initiatives for this, e.g., Raghadan Forest Park, about “burnout prevention” or rehabilitation. There is a forest of educational accompaniment to the patient's chronic pain and mental stress. In this regard, the Research Center for Forests provides impetus by initiating projects that accompany and promote other offers for further training. As a consistent theme, such references in Arabian forestry still need to be anchored (1, 2). The importance of greenery in times of pandemics and chronic diseases is unforgettable. There are still many diseases that can be cured by herbal medicine, and the impact of greenery on the weather is also very important (3–5).

This topic arouses great interest among the Arabian population. The forest's healing effect is described in many research articles, which simultaneously found the healing bond between man and nature, with much focus on nature (6, 7). Interest in the health effects of the forest is present in Arabian society. It is foreseeable that the flow of visitors to the forest will keep growing in the coming years. Without the active participation of forestry, conflicts with forest visitors are predictable, especially since uncontrolled flows of visitors can severely disrupt the forest ecosystem (8–10).

The first step is introducing Arabian forestry to get an overview of important facts. These include the definition of forest, the forest area and its ownership structures, the basic functions of the forest, and the principle of sustainability. In addition, it gives an insight into the importance of forests for the Arabians. The contribution of the research work is given as follows:

- The study aims to show which challenges are posed by the health effects of the forest for Arabian forestry.
- Out of the analysis will be strategic and practical measures for a proactive design of health and forest in Saudi Arabia from the point of view of forestry.
- The focus is on the balance between the health effects of users and the forest, the forest ecosystem, and the economical use of the woods.

## 2. Background

Arabian Forestry represents forest owners whose interest groups include forestry training, further education, and research centers. The health effects of the forest are, on the one hand, health promotion and prevention, such as stress reduction and burnout prevention, and, on the other hand, treatments, such as therapy support for mental or associated chronic diseases. The four fields of activity of the forest are defined in the form of key functions in the forest development plan shown. A guiding function is simultaneously the main effect of the forest and is distributed over the entire Arabian forest area. In 2016, the useful function came in first with 61.7%. The second strongest expression has the protection function with 30.1%. The welfare function increases with 7% of the total forest area in third place. The recovery function comes last with 1.2% (1, 8, 10).

The principle of sustainability is the forest's goal, and the forest law's principle is to be green land. This provision has no direct legal force, but it is essential in interpreting the forest law. The forest law is thus a measure and guideline on which the concrete forest law provisions are based on many orientations. With its effects, the forest is a habitat for people, plants, and animals and is an important basis for social, ecological, and economic development in Saudi Arabia. Sustainable management means caring for the health of the forest and using the forest so that its biological diversity, vitality, regenerative capacity, productivity, and potential are continuously maintained. This ensures the forest has ecological, economic, and social functions at all levels without damaging other ecosystems (11, 12).

The three pillars of sustainability are the economic, ecological, and social functions of the forest that are equally distributed. The primary forest policy mandate of the Forest Act is to balance interests between these three factors. A one-dimensional view of the forest contradicts this principle. In Saudi Arabia, sustainable forest management means contributing to climate protection for the health and vitality of the forest and considering economic aspects. In addition, it also promotes biodiversity, which is a protective function, considering social and economic aspects and international responsibility. The following studies show that the forest is important for any country's population and the Arabian population field (13, 14).

In 2012, the authors established a representative survey by the Integral Research Institute. The focus was on the importance of mountains and forests for Saudi Arabia's identity (15–17). Mountains call for 96%, and for 95% of those questioned, forests remain an essential part of the Arabian identity. Notably, 94% consider mountains and forests important for Saudi Arabia (18–20). The lead in the forest ranking is observed, with 100% seeing forests as part of the Arabian identity. Some other cities have the lowest values at 93%. Mountains and forests also have a high identity factor for young people between 14 and 19 years. In the age group of 60–69 years and those over 70 years, the identity factor is highest at 98%.

The second representative study was carried out by a researcher in 2016. Doing so established that more than 90% of the population regularly goes out in nature. Of note, 73% of those questioned prefer to be in the forest, of which 42% do so daily or several times a week. Approximately 75% of the test subjects regularly spend over 30 years in the forest. Of the under-30 age group, 56% are regularly in the forest and 84% of those surveyed like walking or hiking. For 21%, the focus is on collecting mushrooms and berries. This is followed by the activities such as running and jogging with 17%, observing nature/photographing with 13%, and cycling/mountain biking with 6%. For 69%, the recreational value of the forest is important because they enjoy the silence and are able to recharge their batteries. The average length of stay of those surveyed is up to 1 h for 35 and 45% for two or more hours (4, 21).

The study shows that more people are drawn to the forest, so the pressure to use the forest as a natural space also increases. The forest is accessible for everyone to use, so cooperation works. Notably, 91% of the rules asked for binding rules to avoid conflicts between the different interest groups. The importance of forests, lakes, rivers, and mountains will rise in the future; 75% are convinced of that. Also, the raw material wood itself is gaining in importance for 93% of those surveyed, as this renewable resource is a natural raw material and thus serves to protect the climate (22–24).

The sustainability concept of the Arabian forest is based on four pillars: economy, environment, society, and responsibility toward the following generations. As part of the forest strategy, seven forest policy fields of action cover the following subject areas: climate protection, forest health and vitality, productivity and economic aspects, biological diversity, protective function, social and economic aspects, as well as international responsibility and sustainable forest management. The strategic objectives of action field 6, “social and economic aspects”, are elaborated in more detail as this deals with the health effects of the forest.

## 3. Challenges and developments in the forestry

Looking into the future of forest is essential for economic and social development. To recognize ecological developments and derive a forward-looking one from them to plan and implement forest management. Major trends such as climate change, bioeconomy, urbanization, digitization, and health and recreation should also be related to forestry. These trends and their impact on forestry are discussed.

### 3.1. Climate change

With the result of the project climate scenarios in Saudi Arabia for the first time, the Central Institute for Meteorology has comprehensive data for climate change by 2100. Temperature-related changes will occur by 2050, with an increase in the mean temperature of +1.3°C. The decrease in frost days is up to 2050 between 20.5 and 24.5 days. Similarly, the number of ice days also significantly increases, with daily maximum temperatures of 0.0°C. By 2050, there will be an increase of four to ten hot days (a maximum daily temperature of more than 30°C) in Saudi Arabia (25, 26). There will be significant changes, especially at altitudes below 1,000 m. Concerning precipitation, there are currently no concrete statements. Changes in the total annual precipitation will only become apparent in the future. The climate stress load will generally be high, and the ecological conditions are changing for the forest. The spruce, the main Arabian tree species, comes through endangerment under pressure up to the low mountain range. Harmful organisms are generally a challenge for sustainable forest management. Imports of wood and plants pose a major threat, responsible for introducing non-native insects and fungi, and are favored by the new climatic conditions. The changed climatic influences can reduce the protective effect of mountain forests and reduce natural hazards. Tree species present in the distribution are restricted and will, in the future, also be at home in the mountains (27, 28).

There will also be an increase in the tree line. In silviculture, new and complex challenges are coming to forestry. Silviculture concepts must be considered over a long period of time and simultaneously allow flexibility due to climate change. An essential goal is preserving a site-appropriate variety of tree species in the sense of diversity.

### 3.2. Bioeconomy

Saudi Arabia is oriented toward international climate goals and pushing for an active climate protection and energy policy. There is a reduction in greenhouse gas emissions by 36% by 2030 compared to 2005. An Arabian bioeconomy strategy was decided in March 2019. The goal is to create products that work with fossil raw materials generated by equivalent ones, based on renewable commodity-based. The bioeconomy strategy is the basis of a bio-based economy that should strengthen the Arabian economy's competitiveness. At the same time, a social rethink should be brought about, and greenhouse gas emissions should be reduced (29, 30).

The forest is a key factor in a positive CO_2_ balance. Using wood products, the stored carbon arrives at that time, not into the atmosphere, and at the same time, young trees can absorb the existing CO_2_. Wood is to replace other building materials and fuels in the future. The emissions at production, use, and disposal are lower than those of other building materials, such as concrete or steel, effectively reducing CO_2_ emissions.

### 3.3. Urbanization

Another trend is emerging in urbanization, and 55% of the population lives in an urban area worldwide. By 2050, more than two-thirds, or ~68% of the world's population will live in a city. Cities produce 70% of global greenhouse gas emissions. Due to the rapid development of cities, the danger increases, leading to a climate catastrophe. For these reasons, among other things, urban forestry developed. This form of forestry concerns research and practice dealing with trees, forests, and set-apart green spaces in urban areas. Urban forestry focuses on ecological, economic, social, and aesthetic added value for the city. These include air quality, locations, urban climate, water balance, biodiversity, leisure and recreation, and health and wellbeing. It takes good strategic management and management of the green resource and the involvement of different stakeholders in planning and implementation processes. The growing number of visitors to the exhibition is particularly challenging forest, and the resulting increase in the intensity of use or use activities requires visitor flow control. The networking of the different disciplines and the different interests and demands of the stakeholders are essential tasks within the framework of urban forestry (31, 32).

### 3.4. Digitalization

Digitization means the increased use of networked digital technologies in forestry. The focus is on networking and self-control. Machines and robots are no longer just executors of work steps. By networking all systems, they can make independent decisions about which components go which way in production. Connected to the Internet, digitization is essential for facilitating a quicker exchange of information between suppliers and buyers. Opportunities in digitization include standardization of interfaces, optimization of processes through data analysis, individual product design, and customer-centric thinking. Forestry 4.0 means, among other things, the real forest stock as a virtual forest, ecosystem, and production site. On the one hand, this novel information, planning, and orientation bases are forestry, and, on the other hand, the citizens are looking for relaxation or are made available to tourists. To make forestry competitive, sustainable, and multifunctional, it is guaranteed that in the wood processing industry, the raw material provides wood efficiently (31, 33, 34).

### 3.5. Recreation and health

According to Even et al. (35) and Klingmann (36), health has been less important in many countries. Continuously increases the lifespan of people in better health. The search for strength and life energy is the imperative focus to not go through life permanently exhausted and stressed. These demands have a significant impact on people's lifestyles. Balanced nutrition, physical fitness, individual preventive care, and disease prevention characterize everyday life. Know-how about the human body and its associated health keeps increasing. Scientific and empirical knowledge lead to a new understanding of medicine and thus promote new perspectives and treatment methods (37, 38).

Private individuals increasingly focus on a balanced lifestyle, and companies focus on their employees to create a pleasant working environment through cooperative health. Cooperate health requires clear health management from companies for employees' physical and mental health maintenance. Feeling good increases productivity and sufficient creativity relaxation and is targeted at implementation, leading to a competitive advantage over other companies (39, 40).

Digital networking will completely reshape the topic of health. Open discussions of health issues online, using mobile diagnostic tools and social health movements, will improve communication between physicians and completely redesign patients again.

## 4. Methods

This research consists of a theoretical and an empirical part. The theoretical part depicts the current scientific view to answer research questions. It gives an overview of Arabian forestry, forestry strategies, development and challenges in forestry, and the importance of the recreational effect of the forest. In addition, the health issue is connected with the discussion of the forest as a recreational space. The connection between forests and health is outlined as part of the topic “Green Care”. As a result, the health location of forests and its effects are shown.

The empirical part of the work includes two research methods to approach the answer to the research questions. In the first step, a qualitative research method was chosen. Expert interviews (see Appendix 1) with responsible persons from forestry were conducted in this context. In doing so, their view on the topic of “health effects of forests” was shifted. In the second step, quantitative research was carried out. Many students were asked about their usage behavior of the forest using a questionnaire (see Appendix 2). In addition, questions were asked regarding their awareness of forests and health. Based on the scientific basics and empirical information, measures for Arabian forestry are formulated as recommendations. This course is a critical examination of the knowledge gained from the work.

The interventions to implement the health effects of the forest aim, on the one hand, to promote health and, on the other hand, to improve mental, physical, emotional, and behavioral problems. Among those were prepared by the expert group “Forest for human health and wellbeing”, which defines four implementation categories:

i. *Forests for health promotion and disease prevention*: this approach includes medicinal forests, medicinal forest paths, regeneration, and wellness paths implemented. In addition, include mindfulness programs and offers for forest bathing and stress reduction.
ii. *Forests for therapy and rehabilitation*: within this framework, combinations of nature and psychotherapies, forest therapies or forest therapy hikes and rehabilitation, and forest and social integration.
iii. *Forests for direct and indirect health education*: these concepts imply educational outdoor programs such as field trips, thematic excursions, workshops, observation trips, forest schools, and forest play groups.
iv. *Forests for tourist recreation*: these include guided hikes, themed excursions, bridle paths, mountain biking, nordic walking, forest vacations, orientation programs, forest museums, etc.

Based on the theoretical principles described so far, the next section presents the empirical research work to link them together and gain new insights.

## 5. Empirical research work

Qualitative and quantitative analysis is performed to support the research work. The following research questions are derived.

1) What developments and challenges in Arabian forestry are foreseeable in the next 10 years?
2) What are the forest's potential health effects for Arabian forestry in the coming 10 years?
3) (A) What effects do the users of the health effects of the forest have on the ecosystem and the economic use of the forest? (B) Which main user group do the representatives from the forestry represent concerning the health effects of the forest now and in 10 years?
4) Which wishes/needs about the health effects of the forest are there from the user's point of view?

To be able to answer the research questions, two different methodological research approaches were chosen. In the first step, a qualitative research method was adopted as the main method used to provide information. In this context, expert interviews with representatives were conducted. The second step was a quantitative research method as a secondary method to obtain information. For this purpose, a written questionnaire survey with medical students was conducted.

### 5.1. Qualitative research

Qualitative research is carried out through expert interviews. The expert interview was chosen as the method of qualitative research. Experts are people who have specific, required role knowledge. This is also attributed to them; thus, they have a special competence. This knowledge includes operating knowledge (e.g., processes, rules, and connections), interpretation knowledge (e.g., as an actor in a certain field of discourse), and contextual knowledge about other participants in the investigation. The expert interview takes place in the form of a guideline interview. The expert interviews were conducted based on a few parameters: creation of the guide and implementation of a pre-test, selection of experts, contacting the experts regarding availability and appointments, carrying out the ten expert interviews, transcription of the expert interviews, and evaluation of the expert interviews.

Since the question of the research work from the forestry perspective is illuminated, forestry representatives were selected. They included the following criteria: knowledge of forestry, goals (e.g., processes, traditions, connections, and developments in Arabian forestry), mapping of different ownership relationships, integrating forestry research and educational institutions, and individuals considered network nodes in the forestry context.

The data were collected through a guided interview. The guide is a structured, written catalog of questions and serves as an orientation during the interview. It contains all the important questions and provides at least a rough structure. The guide is based on three parts: the introductory question; the main part with blocks of questions, topics, and subtopics; and closing and thanks. The interview guidelines within the framework of the expert discussions were based on the research questions (see Appendix 1).

The interviews were then transcribed. The transcription form used depends, on the one hand, on the research methodology and the expectations of knowledge from time to time, as well as on research pragmatic additions. Therefore, it must be clarified in advance what the transcript is being created for and how the analysis should be set up. When transcribing, it needs clarity on what should be written down and the rules based on that. This helps get the job done scientifically to maintain the common thread when reading later loses.

The evaluation of the collected data was carried out according to the methodology of the content-structured qualitative content analysis, as described below, in the context of the master's thesis. The categorization and coding were done in a single, multi-stage process. The main categories were formed deductively by adopting them from the interview guide. A second or third revision defined the subcategories in a mixed form. The main categories differentiated subcategories were partly from the interview guidelines adopted (deductive) or are new in the evaluation context (inductively)—the category-based evaluation gained during the evaluation by comparing and contrasting sophistication, complexity, and explanatory power.

### 5.2. Quantitative research

The quantitative research approach is object-related and tries to identify explanations and cause-and-effect relationships within this framework. A feature of quantitative research is to deal with social reality through objectively controllable methods. The aim is to define behavior in models, describe numerical data, and make it predictable. We collect data that meets and serves the criteria of objectivity, reliability, and validity to test theories and hypotheses. As a quantitative research method, a written survey using a standardized questionnaire. The survey is very common. It is mainly used when human behavior is not over, and the observations can be recorded, but the test person must provide information themselves. This includes opinions, attitudes, and human behavior. Characteristics of the written survey are:

- The test person needs a personal counterpart when filling out the questionnaire form.
- There is a temporal and spatial distance between the interviewer and the subject.
- The interviewer cannot provide any support in answering the questions permitted.
- There is no certainty that the subject will fill out the questionnaire.

The author follows the following sequence to develop the surveys: creation of the questionnaire, definition of the target group, carrying out a pre-test, revision and finalization of the questionnaire, commissioning a student to carry out the survey, and evaluation and interpretation of the results. The author originally planned the target group of the quantitative survey to collect the “health effects of the forest” within the framework of the expert interviews. The experts' perspectives on who could be a user group were very wide. The general feedback was that potential users of the forest are all humans, and therefore it was impossible to determine a specific target group on this path. However, within the framework of expert interviews, the medical field is often seen as a potentially more interesting future cooperation partner. Therefore, based on the following criteria, the target group of the quantitative survey is defined as potential users of the health effects of the forest, potential future cooperation partners for forestry, and people in a medical facility very close to a recreational forest. The feedback was incorporated, and the questionnaire “Health Effects of Forests” (see Appendix 2) was implemented. In the survey of 2,500 people interviewed, 64% were male and 36% were female between the ages of 23 and 32.

## 6. Results

### 6.1. Results of qualitative research

The results of the interviews with the experts are presented below using the five main categories shown: general challenges for Arabian forestry, developments in the area of forest and health, potential for forestry, users and their impact, and strategic and practical actions.

#### 6.1.1. General challenges for Arabian forestry

When evaluating the interviews, four main topics emerged as the future general challenges of forestry emerged.

##### 6.1.1.1. Climate change

Climate change was a topic in all interviews and is referred to as “the” future seen in the challenge of forestry. In the course of climate change, the forest at the same time affected an essential shaper, e.g., the reduction of CO_2_ (emissions), or it served as a compensation factor for temperature differences. It will be certain that this change will come, but its reactions will be different. Some of the forest owners are waiting and working as before. At the same time, science is expected to be more well-founded shortly. To be able to provide insights into climate change and develop clear solutions. This means site-appropriate mixed forests tolerate the drought well, especially in the lower altitudes. Some establishments are ongoing due to calamities caused by the bark beetle, which has already started with the conversion and has a reduced proportion of spruce. There is also some helplessness in the forestry sector, especially in companies that have always practiced near-natural forestry and still have suffered major damage from storms, broken ice and snow, and drought.

##### 6.1.1.2. Economic challenges

Climate change is also economically relevant for forestry. The forest owners must watch out for damage caused by bark beetles or extreme weather events that react quickly. Nevertheless, two experts also critically questioned whether or not their own omissions in management contribute to the current economic situation. The recurring catastrophic events strongly influence the wood market. The price of wood is subject to fluctuations that follow. In addition, forestry generally sees itself as having stagnating wood prices and constantly rising wages. Many forestry companies traditionally live from selling wood and hunting, causing them great distress. Diversification in the forestry industry would be important in the future so that forest owners from the timber industry are not entirely dependent.

In addition, the social benefits of the forest, as something taken for granted by society and taken for free by the forest owners, must be made available. Forest companies invest in the company without receiving anything in return, and as the quote below shows, this should change in the future.

#### 6.1.2. Developments in the field of forest and health

In the context of the expert interview, the developments in Arabian forestry were also asked. The most important topics presented.

##### 6.1.2.1. Forestry

Different approaches are currently recognizable under the term Green Care Forest. On the one hand, the health effects of the forest are explicitly understood. On the other hand, the four subject areas of forest education, forest and culture, forest and tourism, and health effects of the forest are subsumed under this term. Few experts had concerns about the time and money forestry would have to invest in establishing itself in the healthcare market. Traditional thinking strongly influences forestry and reacts to these “new” developments in a wait-and-see manner.

##### 6.1.2.2. Collaborations

The health effects of the forest can be established through forestry cooperation. The potential is seen in four possible areas of cooperation:

i. Health sector: possible cooperation partners would be, e.g., doctors, therapists, special houses, and clinics.
ii. Leisure time: the users are direct cooperation partners, and appropriate projects are developed together.
iii. Tourism sector: potential is in cooperation with large hotels or tourism regions, especially regarding quality tourism.
iv. Public institutions: shared mountain bike trails or bridle paths are available.

The use of the forest roads is financed by public institutions that are supported and assumed insurance about liability. Region-specific solutions are required. Four experts have rejected public funding because this will make it worse, restricting the right to self-determination of the owner.

### 6.2. Results of quantitative research

The results are clustered according to the topics: stay in the Forest, Activities in the Forest, Effects of the Forest, Requirements for Wellbeing in the Forest, and Awareness of Forest and Health.

#### 6.2.1. Stay in the forest

A stay in the forest was associated with nature by 27%. Included trees and animals played the leading roles. In addition, for example, our own closeness to nature, special smells, and forest noises. The movement is another point of association with the forest for 21% of those surveyed. Walking, running or jogging, and hiking were the most important foregrounds. In addition, cross-country skiing, downhill skiing, and cycling were reported. Other categories were the seasons with 8%, social with 3%, and others with 4%.

#### 6.2.2. Activities in the forest

The test subjects did the following activities: being in the forest, walking in the forest, hiking in the forest, meditating in the forest, doing sports in the forest, working in the forest, and relaxing in the forest. They could answer the following possible questions: daily, several times a week, several times a month, less often or never, or no information. Derived from the median and mean, as can be seen in Table 1, the most common activities of the test subjects are (1) to be in the forest, (2) to relax, (3) to exercise, and (4) to go for a walk. Hiking, meditating, and working are activities that are less practiced in the forest.

**Table 1:** Frequency of carrying out activities in the forest.

**Item**	**Median**	**Average**
I'm in the forest	2	1.66
I relax in the forest	1	1.30
I do sports in the forest	1	1.29
I go for a walk in the woods for an hour or two	1	1.20
I go hiking in the forest for half a day or more	0	0.80
I work in the forest	0	0.27
I meditate in the forest	0	0.10

Annotation: 0, never; 1, less often; 2, several times a month; 3, several times a week; 4, every day.

**Figure 1:**
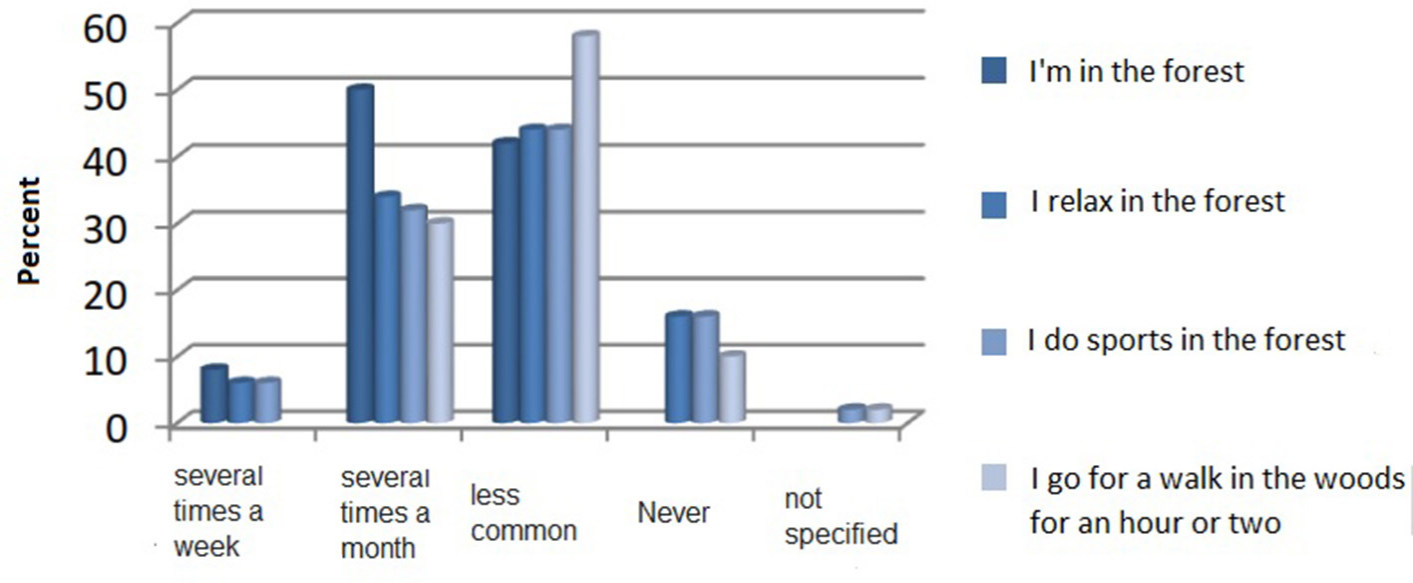
The four most common activities carried out in the forest in percentage.

Figure 1 shows that 50% of the test subjects are in the forest several times a month, 42% are less often in the forest, and 8% are several times a week. None of the test subjects visit the forest on a regular basis or at all. The topics of recreation and sports show similar residence patterns. Approximately 34 and 32% hold on to this several times a month in the forest. Notably, 44% of those questioned held themselves to this more rarely on-site in the forest. For 16%, neither sport nor recreation is an activity that exercises them in the forest. Approximately 6% of the respondents do this activity several times a week in the forest. Nobody goes to the forest every day for recreation or sport. Approximately 30% take time to walk several times a month, and 58% of those surveyed walk in the forest less often. Of note, 10% never go for a walk in the woods. None of the subjects go for a walk daily.

**Figure 2:**
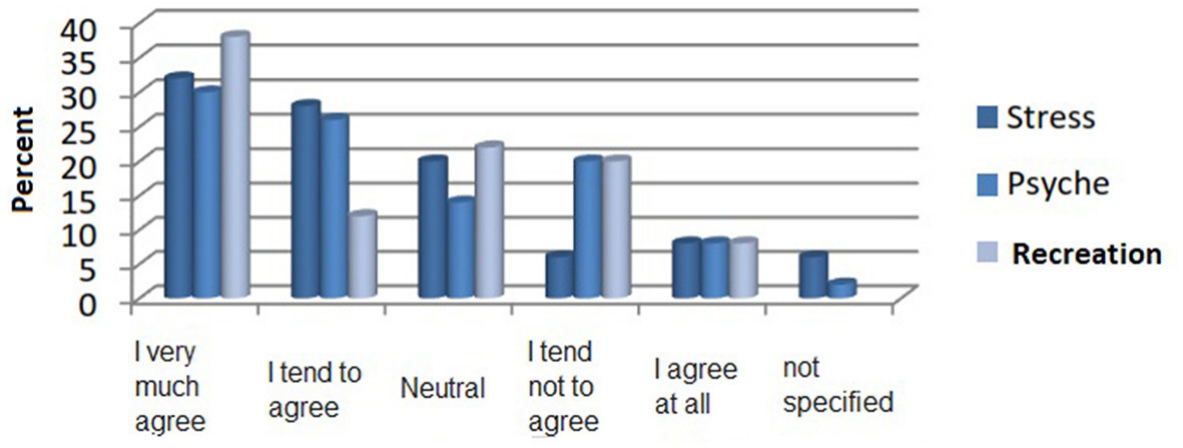
The assessment of the positive effects of the forest on stress, psyche, and recreation in percentage.

As seen in Figure 2, the first three in a row decelerate in stress, positive for the psyche, and, more importantly, recreation room an agreement of very or the possible answer of more than 50% of subjects. Interesting differences between the male and female subjects are in the subject areas of effect on wellbeing and effect on health. A very or rather positive effect of the forest on general wellbeing, ~37% of the male test persons agree, and 34% see this neutrally. In contrast, approximately 66% of the female subjects agree very much or tend to agree, and only 11% have a neutral vision. The results of the Mann-Whitney *U-*test show that the information between men and women does not differ significantly on average from each other (*Z* = −1.505, *p* > 0.05). The calculations of a Mann-Whitney *U*-test (41) show significant average differences between men and women in their statements about the effect of the forest on their health (*Z* = 2.052, *p* < 0.05). Women vote for the health effects of the forest more than men. A high impact on the forest is healthy in ~66% of the female test subjects and ~27% of the male subjects. Approximately 44% of the male subjects tend not to see the importance of the forest for their health, and 11% of the women agree.

#### 6.2.3. Requirements for wellbeing in the forest

When asked the subjects what was particularly important to them during their stay in the forest, 14 subject areas were queried, ranging from 1 = very important to 5 = not at all important or no information could be evaluated.

**Figure 3:**
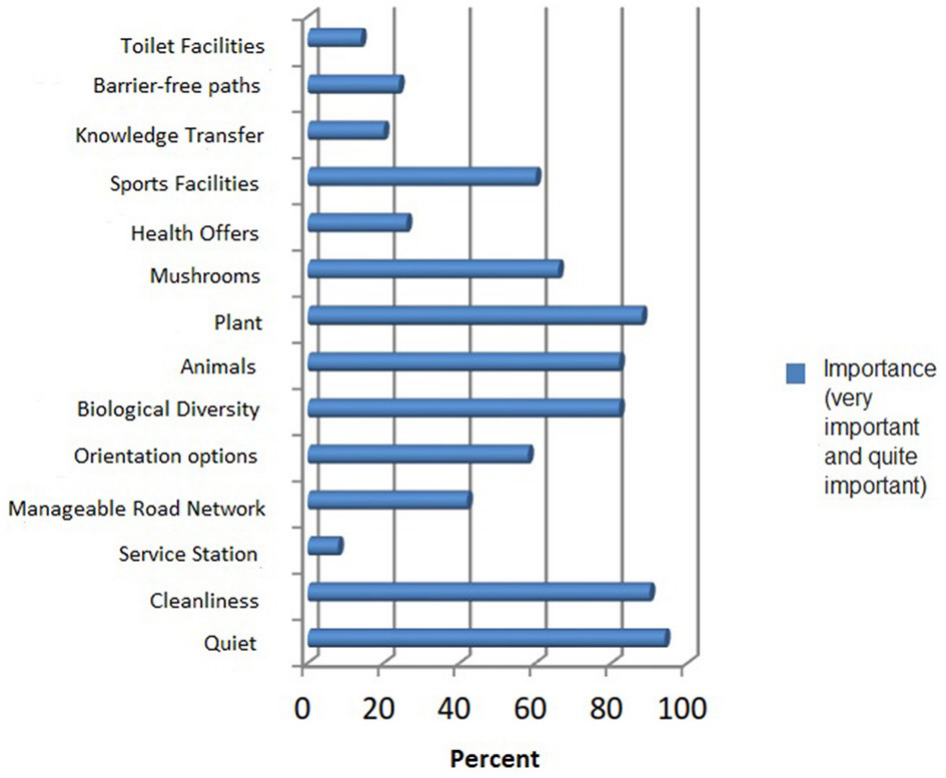
Significant elements when staying in the forest in percentage.

Figure 3 shows that cleanliness in the forest subjects is most important. For example, 90% of those surveyed rated this as very important (80%) or fairly important (10%). Closely followed by the rest, this one was very important to 66% of respondents and fairly important to 28%. In a row, then stood for the subjects—the fauna and flora of the forest and their diversity—on the importance scale at the top. The sports facilities in the forest are also important.

These were rated as very important or fairly important by 60% of the subjects. The importance of infrastructure, such as orientation options and a walkable network of paths, was ranked in the middle. Barrier-free paths, knowledge transfer, rest stops, and toilet facilities are used by a quarter or classified as less than very or fairly important. Health-promoting offers are considered very important or fairly important by 26%.

**Figure 4:**
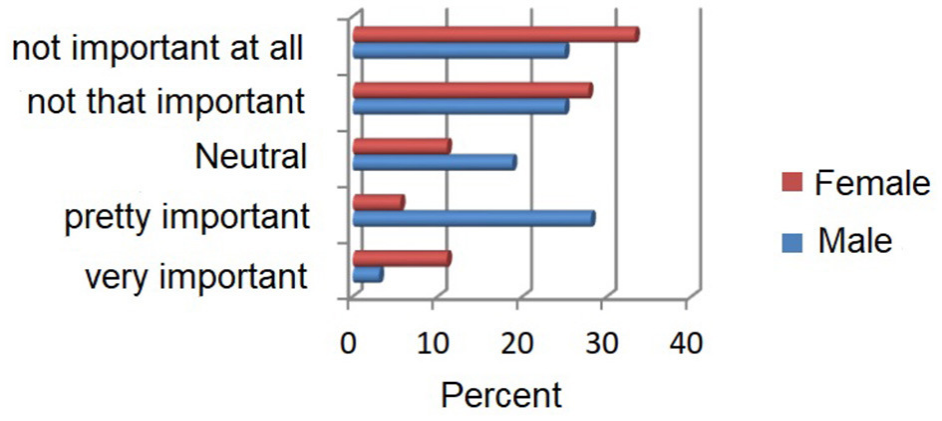
Importance of health-promoting offers in percentage, separated by gender.

Figure 4 shows an evaluation of the health-promoting offers separately by gender. Approximately 30% of the male subjects estimate health-promoting offers as very or fairly important. See this in Only 17% of the female subjects compared this way. The results of the Mann-Whitney *U-*test show that the differences in the information averaged between men and women are not significant (*Z* = −1.003, *P* > 0.05).

#### 6.2.4. Awareness of forests and health

The last three sections of the survey were devoted to the health effects of forests. In the first step, general information is obtained to determine to what extent the subjects are already familiar with this topic. The response scale ranged from 1 (i.e., very much) to 5 (i.e., not specified at all). To have an equal starting point, it was first defined that what goes into this survey is to be understood as the health effect of the forest. Health effects include health prevention, such as burnout prevention, strengthening of the immune and cardiovascular systems, and other treatments, such as therapy support for psychological or chronic diseases.

**Figure 5:**
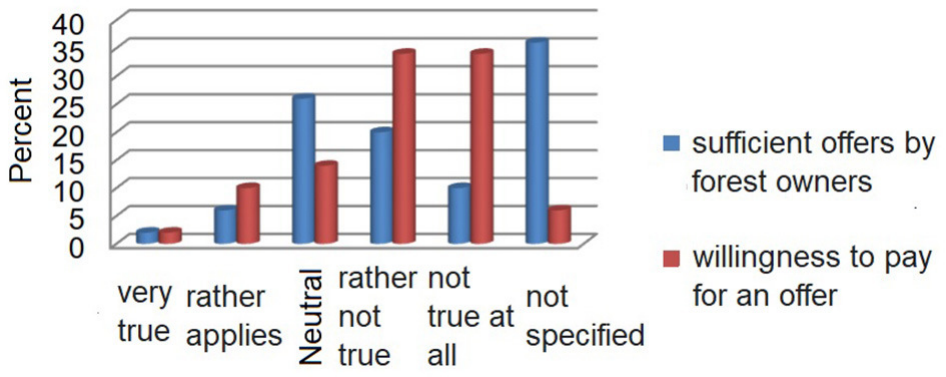
Knowledge about the health effects of forests and offers in percentage.

More than 50% of the test subjects thought that raising awareness would be important in terms of the health effects of the forest on society, and this item received the highest agreement. Approximately 40% believe that the topic of the future in Saudi Arabia is gaining importance. Just under 20% of those surveyed feel well-informed on the subject. About a quarter of those surveyed would like to join in the future to deal more with this topic, with 38% being neutral on the topic to face. Only 8% of the test subjects consider the offers of forestry in this field sufficient. Notably, 36% could not provide any information at all; 2% have already paid for such an offer; or 12% have, in principle, a willingness to pay for it. Figure 5 shows a comparison of the following items: “In principle, I would be willing to accept the offers pay” and “Arabian forest owners provide sufficient in this regard offers available.”

Figure 5 clearly shows that, on the one hand, there needs to be more knowledge about the offers from the forest owners, or these cannot be assessed. However, it is also evident that the willingness to pay for such offers could be higher. In the thematic block “Forest meets medicine”, what perspectives and knowledge could the subjects answer with 1 (i.e., very much applies) to 5 (i.e., applies not at all or no information) given? Almost 88% of respondents said they knew none, and ~6% were neutral and 4% of the subjects made no statement on this. The statement, “I believe that the forest plays a major role in health prevention for the population,” gifts 46% of the respondents very or. Rather their approval, 40% are to the theme across from neutral, and 10% were unconvinced. In addition, 4% could make no statement. For the item “I think the health effects of the forest can be medicinal support treatments,” ~70% of the test subjects were very or rather convinced. Notably, 12% of those questioned were not convinced or not at all convinced, whereas 16% had a neutral approach and 2% gave no feedback.

**Figure 6:**
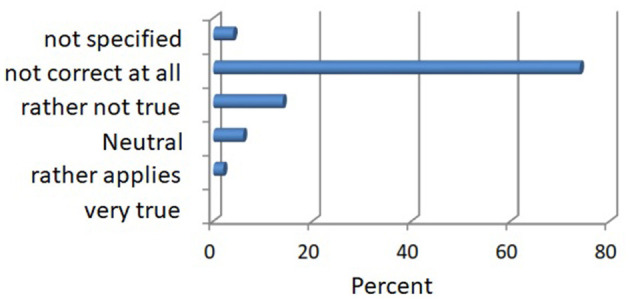
Knowledge of scientific studies on the topic of the health impact of the forest in percentage.

In the thematic block, “Forest meets medicine”, with all the perspectives and knowledge. The subjects (survey form) could answer in a range from 1 (very much applies) to 5 (applies not at all or no information). The results of the surveys are given in Figure 6.

On the topic, “I am aware of scientific studies on the health effects, as per the surveys”, 2% of those questioned stated that they were aware of such studies. Almost 88% of the respondents said they knew none, and ~6% were neutral while 4% of the subjects made no statement on this.

**Figure 7:**
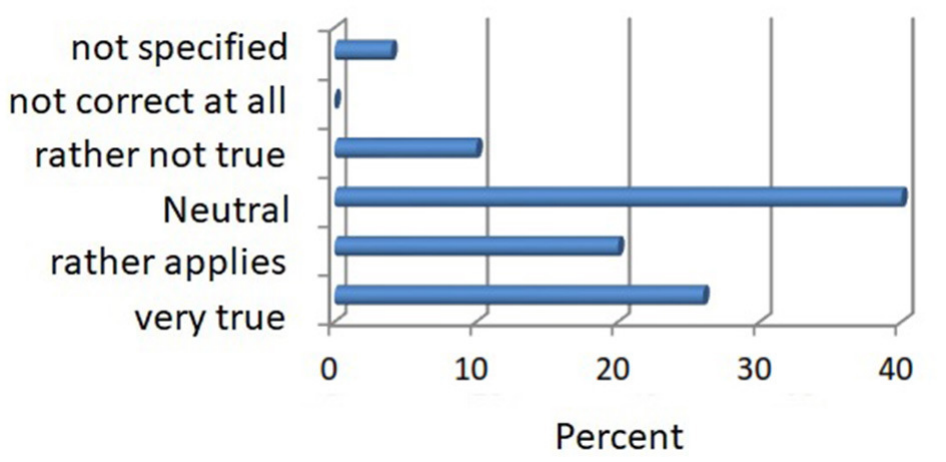
The role of the forest in health prevention in percentage.

For the other statement, “I believe that the forest plays a major role in health prevention of the population”, 46% of the respondents were neutral, 30% were rather applied, 40% approved it as true, and 10% were of that rather not convinced, and 4% could, in addition, make no specific statement. The survey data are given in Figure 7.

That the forest in the vicinity of a clinic affects the wellbeing of the patients promotes, 80% of the test subjects agreed very much or rather; 12% had one neutral opinion; only 6% tended to disagree; 2% were able to do this, giving no information; 78% of the test subjects are very or somewhat of the opinion that forest owners' interesting cooperation partners for medical facilities such as rehabilitation, psychiatry, or health resorts would be; 10% of respondents had a neutral opinion; 8% tended not to share this opinion; and 4% said there was no specification. At the end of the written survey, the subjects were asked to give wishes to the forest owners about the health effects of the forest. Notably, 55 (=100%) different responses were given; these have been broken down into five main categories:

- A large part of the wishes was aimed at the protection and natural conservation of forests, with 38%. These include topics such as promoting biodiversity in the forest, maintaining the forest sustainably, reducing management, and protecting the nature of the forest.
- Another category important to the test subjects is 24% information and communication on the health effects of forests. In addition, there were requests for more information and public relations, clearer offers, concrete cooperation in therapy, and the desire that forestry also expresses what it would need from the population.
- The third category, with 24%, concerns opening up the forest, including, among other things, wishes expressed, such as lifting the driving bans, more sports facilities, and expanding public access to the forests.
- The cleanliness and tourism categories each had 7%. The topics of cleanliness were more cleanliness, improved waste disposal ideas, and litter prevention. Regarding tourism, two thematic areas were forest tourism (primarily motocross and helicopter) and prohibiting or avoiding excessive tourism (mainly for rest, animals, and recreation).

## 7. Summary of key findings

Up to 47.6% of the Arabian area is covered with forest. A special feature of Saudi Arabia compared to other European countries is that a high share (82%) of private owners exist. Forest development plans are important instruments for forest policy and forest law decisions. Here, the recreational function is limited, with only 1.2% of the Arabian forest areas expelled. The principle of sustainability is the goal and principle of the Forest Act and thus the measure and guideline for its interpretation. The three pillars of sustainability are forests' economic, ecological, and social functions, which have equal rights. The forests have a high identity, creating importance for the Arabian population, with young and older people feeling closely connected to the forest. People are increasingly drawn to the forest, and as a result, higher usage pressure is to be expected in the future.

The United Nations 2030 Agenda is an action plan for planet Earth, and the forest strategy developed as part of this is based on a sustainable approach to forest management (42). Here, too, there is a balance between the economic, ecological, and cultural (social) values of all kinds for present and future generations. At the European level, 40% of the land area is covered with forest. In contrast to the global decline in forests, the Middle East's forest area is increasing easily. Within the framework of the principle of subsidiarity, each country in the Middle East determines its forest policy.

The field of action “Society and economic aspects of the Arabian forests” of the forest strategy are defined as strategic objectives on the subject of the health effects of the forest and give suggestions for strategic measures. This includes, for example, raising awareness about the health effects of the forest, the reconciliation of interests between forest owners and social groups, and health programs on all levels. For silviculture, this means close-to-nature forestry in the longer term, thinking in terms of time, and yet being flexible for the unforeseeable. Another change coming soon is the bioeconomy. Fossil raw materials should go through renewable raw materials to be replaced. One source for this is the forest, which could replace previous building materials and fuels. This is another development Urban forestry theme. In 2050, 68% of the world's population will live in cities. Urban forestry focuses on creating ecological, economic, and social values and generating aesthetic added value for the city. Also, the topic of digitization will keep forestry busy in the coming years. The virtual basics can be used by citizens and tourists looking for relaxation. Recreation and health are another big trend happening in society, and forestry will have an impact. People are looking for strength and life energy, which are also found in the forest.

The Arabian Forest Act stipulates that every person can use the forest for the purpose of recreation. Different values and increasing societal demands on the forest and its effects are breeding grounds for conflicts between user interests. Missing numbers and facts related to leisure and recreational activities in the forest also make good control difficult. It will be integrative and require forest management to prevent conflicts in advance. Liability issues are also future focal points concerning opening forest roads for various sports, such as mountain biking. Because of nature sports, the pressure on the ecosystem increasingly holds people around the clock in the forest.

The ecosystem services of the forest are geared toward human wellbeing. The central question arises as to how society this added value provided by the forest owners will be able to compensate in an appropriate form. The WHO describes health as perfect physical, mental, and social wellbeing (43, 44). The Arabian health goals are derived from Agenda 2030 and the Health 2020 concept. The 2030 Agenda includes Goal 3 to ensure a healthy life for all people of all ages and promote their wellbeing. The Health Concept 2020 is about improving health and reducing health inequalities.

Contact with nature does not automatically mean Green Care health-promoting interventions with animate or inanimate nature give Green Care its meaning. The aim is to maintain and promote social, physical, and mental wellbeing. International Green Care includes horticultural therapy, care farming, animal-assisted therapy, and natural therapies. Saudi Arabia developed its own Green Care strategy. Green Care Forest is part of Green Care Saudi Arabia and supports people's mental and physical health to maintain, increase, or restore. Green Care Forest is based in Saudi Arabia on four pillars, namely, forest education, forest and culture, forest and tourism, and forest and health. There are legal framework conditions for health offers in forest observation. The main influencing factors on the health effects of the forest are the duration of stay, the type of landscape, the forest aesthetics, the social context, the psychological activities, and possible negative influencing factors. The Health Place Forest has a health-promoting, preventive, and therapeutic effect on people's physical, mental, and social health.

This results in a stress reduction, a strengthening of the immune system, the prevention of cardiovascular diseases, an improvement in sleep quality, and a strengthening of psychological wellbeing and social integration. A qualitative method was used for the empirical research work based on this in the form of expert interviews with representatives of forestry, who also represented the main method simultaneously. As a secondary method, a written questionnaire was used for medical students.

### 7.1. Findings from the expert interviews

The general challenges of forestry in the next 10 years are linked to climate change, for which there are still no definitive solutions, and the resulting economic developments. In addition, there will be areas of tension between forestry and the requirements of a strengthened society. International and national strategic fields of influence for forest-related strategies will also affect forestry in the coming years.

Regarding the health effects of the forest, forestry and the population perceive very different developments. Green Care Forest will be strong with the recreational function connected; therefore, an economic value currently needs to be discovered for forestry. In contrast to forestry, forest and health care in society are perceived as a strong trend by the media, and popular literature is additionally promoted. Potential is also seen in the health effects of the forest. So it will be assumed that this topic will be an image boost for forestry. It is also recognizable that the health effect of the forest is greater. Product variety for forestry is possible, and thereby, an additional source of income could develop in the form of a niche product. Cooperation would be particularly interesting for the health sector. They will also see an economic benefit since the forest's health effects generally promote or support people's health.

Potentially everyone will be the main user of the health effects of the forest people see, whereby the specific offers must be worked on in a target group-oriented manner. The impact of users on the forest ecosystem is classified as low, provided there is good visitor management and communication. Hunting is an exception to this, as are possible conflicts of interest in this area. The users' impairment in economic use is rated much more strongly. The romantic “transfiguration” of the population always arises, especially when using the wood for bigger conflicts again. More users mean more, while stronger security measures and control rounds increase responsibility.

For a balance between users, the forest ecosystem, and the economy, the following strategic and operational measures should ensure use. One of those is the establishment or expansion of professional visitor management. Next is public relations work to better show the forest owners' services in representing society. The reconciliation of interests will also become an issue in the future important strategic package of measures. This requires cooperation with users and with non-profit organizations. Obtained through urbanization, the products within the framework of the health effects of forests have a chance on the market. It is important to present the added value beyond the recreational effect. Awareness raising and clarification on the subject are also needed internally. The forest owners must be aggressively involved in product development. The research and educational work on forests and health is still in progress in the pioneer phase. There are some initiatives in the research field, but only some result summaries. Networking and various cooperatives are sites of forestry that are especially important in the different regions.

### 7.2. Findings from the written survey

When asked about a spontaneous connection with the last visit to the forest, “recreation and health” is the top priority. Followed by the second place with the keyword “nature” and the third place with “movement”. In the activities practiced in the forest, it is most popular “to be in the forest”. This is followed by “recreation” and “sport”, also practiced with pleasure. For both positive effects of the forest, the top priority is “deceleration in times of stress, strengthening of psychological wellbeing”, and “recovery”. The positive effect on “health” is significantly stronger in the female subjects perceived than in male subjects. Pristine forests, mixed forests, and open sparse forests are preferred for a stay in the forest. Monocultures, as well as strongly managed forests, are less in demand.

Awareness raising about the health effects of the forest is considered very important by the subjects. Almost 40% believe this topic will gain importance in the future. Only a fifth of respondents feel informed about the subject. Only very few subjects could assess forestry offers on forest and health handover. At the same time, there needed to be more willingness among the subjects to accept such offers. Only one test subject was allowed to conduct scientific studies on this topic. Almost half of the respondents believed that the forest in preventive healthcare plays an important role. More than two-thirds of respondents believe the forest's health effects support medical treatments. Just as many assume that a forest in a medical facility's environment promotes patients' wellbeing. Two-thirds of the test subjects also see forest owners as interesting cooperation partners in the medical field. When it comes to the wishes of forestry about the health effects of the forest, the protection and preservation of natural forests are ranked first. However, the wish came true after more information and communication. Peer was also wishing for more opening of the forest for visitors.

### 7.3. Interpretation of the findings

The Arabian Forest Strategy 2020+ was created as part of a broad-based participatory process. This enabled the integration of many different stakeholders and is considered a showcase project in terms of participation. The health effects of the forest are made explicit, in contrast to the international strategies mentioned in the Arabian forest strategy, and testify to innovation potential. At the same time, the strategy is also a compromise. Many strategic subject areas and measures will be named without clear responsibilities and time frames for implementation. As a result, the strategy loses traction and liability for the responsible stakeholders. In addition, the question defined indicators. The number of projects says nothing about raising awareness of the importance of the health effects of forests. The impression was created that the forest strategy, which is a very good instrument, needs to be more relevant to practice in forestry, which is necessary for a strategy.

The term Green Care is strategically and in terms of content strongly influenced by agriculture. Green Care Forest still needs to have a clear profile in forestry. It means different things at different levels. So is what the Federal Ministry for Tourism and Sustainability understands by this forest education, forest and culture, forest and tourism, and forest and health. As part of Green Care Forest, the Federal Research Center for Forests relies on the classic definition and understands the educational, health-promoting, and social activities and interactions between humans, animals, and nature. As part of Green Care Forest, images that only deal with forests and health set the focus. These different interpretations indicate a missing coordination process between the different responsible parties. In addition, it is unclear who is responsible for this topic, as the so-called “process owner” pulls. A successful brand establishment needs a clear profile with a clear definition and objective, and a responsible person who drives the brand forward.

The forest development plan is an important forest policy instrument in Saudi Arabia. As part of this, only 1.2% of the area is designated recreational forest. Here it becomes clear how unattractive the recreational forest is for the Arabian forest owner. The Forestry Law of 1975 opened a door in Saudi Arabia. All forest owners are obliged to admit people to their forests for recreational purposes without receiving any financial compensation. The tendency of forest visitors is increasing, and with it, the target group's requirements. The question of liability has yet to be satisfactorily clarified, and forest owners are in danger of being sued. This causes forestry rather than defense mechanisms to develop. The tension between private property and public interest comes to bear here. For forestry, the question arises as to what else is additionally reasonable. In the context of the expert interviews, this question was repeatedly discussed.

Both international and national forest management strategies have one focus on sustainable forestry. This means managing the forest in such a way that, currently and in the future, the forest's economic, social/cultural, and ecological functions are secured. This goal definition runs as a red thread through the research work. Economic, ecological, and social goals are often divergent and carry the potential for conflict. In the future, it will need more participatory ways in the form of cooperation, joint projects, and decision-making processes with the different stakeholders. Balancing interests thus becomes an important management task. In addition, it takes courage in forestry; these new ways of working together try out because they also have much innovative power. Due to climate change and the resulting disasters such as storms, frost, and drought, the economic situation of forest enterprises is very challenging. It follows unplanned high costs to reforest and maintain the areas.

In addition, the price of wood is stagnating or is subject to negative fluctuations. Traditional sources of income from timber production and hunting often need to be increased. In both expert interviews, it was mentioned again and again that there are additional mainstays needed to be able to survive economically in the long term. Such a foothold could be one of the health effects of the forest. The added value of a “health place forest” for forestry is not yet tangible or visible. The previous pilot projects show the first possibilities on a “small” basis but are mostly financially unattractive. They find many imitators in forestry. In an economically difficult situation, building something new simultaneously requires much courage and an attractive vision, which still needs to be developed for the health effects of the forest, among other things. Many forest companies also need more resources and know-how for their product development.

The forest ecosystem is severely affected by climate change. A climate-friendly forest will primarily be associated with a silvicultural conversion of the forest, and diversity is seen as an important success factor. More people in nature and all that around the clock also put greater pressure on the different ecosystems in the forest. How visitor flows affect this currently needs to be assessed. Visitor monitoring concerns forest visits in Saudi Arabia, limited to national or nature parks. There needs to be more data and facts on how forest visitors affect the regions, e.g., in urban areas or tourist areas. As a result, an important basis for argumentation about politics and society is to enforce forestry interests.

Research on the “health effects of the forest” is available in Saudi Arabia but is still expandable. In the expert interviews, it became apparent that within the framework of the training sector in forestry, there needs to be more knowledge on this topic. The exception here is a certificate course, “Green Care Forest”. Parallel to forestry, a new education market is emerging, e.g., forest bathing or mindfulness training in the forest. Forest health education is pure knowledge transfer and therefore does not require a qualification. This means that the education market is about forests and health and is open to all people who feel qualified to do so. In the medical sector, as the survey of medical students became visible, hardly any knowledge was conveyed about the health effects of the forest. So that the topic of forests and health can be seriously brought to society, more investments are needed in education, including state-recognized training standards. Forestry cannot do this alone, but only by implementing cooperation, e.g., with the health sector.

Potentially everyone will be the main user group for the forest as a healthy location people perceived. This has the advantage that a broad target group can be addressed here. Individual forest owners will focus on certain groups of people who need to specialize to market themselves in a region. Cooperation partners will be needed for larger projects like establishing a healing forest. Since the forest ownership structure in Saudi Arabia is very fragmented, larger projects are becoming more common. Setting goals can be a big challenge. Become a cooperation partner on the part of the forest experts in the medical and soft tourism fields. In addition, it should also be mentioned that the medical students, in the context of the written forestry survey, are also an interesting cooperation partner to imagine. In addition, companies would also be interesting cooperation partners, as they are interested in Cooperate Health to keep their employees healthy.

Health has a high priority in society itself, and the trend is going in the direction of self-optimization of overall health and the search for strength and life energy. This search will strongly impact people's lifestyles in the future. The forest as a health location has many prerequisites regarding the vision of the future WHO. The Arabian health goals are also contrary to the forest's health effects. The health sector's trends, visions, and goals have less of an eye on forestry. But they have potential, for example, in the context of digitization. In forestry and the health sector, digitization will take hold to a large extent in the next 10 years and completely realign previous work. New synergies can be created here, e.g., in the form of apps between the green and white areas. It was interesting that the medical students started their last stay in the forest, which connected health and recreation in the first place. So at the test, people have a positive experience, and they have the connection between the forest and health produced intuitively.

In the specific questioning about the effects of the Waldes, primarily stress reduction, strengthening of psychological wellbeing, and recovery receive a high level of approval. This results in a similar picture as in the scientific studies on the health effects of the forest and corresponds to the trend toward the search for strength and life energy. It is striking that, especially for female subjects, the effect of the forest on health has a higher force attribute. The Mann-Whitney U test shows significant results. This matches with the scientific evidence that women, on the health effects of the forest, react more strongly than men, especially in the context of stress reduction.

In a survey of an older target group, they would probably have infrastructure facilities or a walkable network of paths that have a different meaning than for the young medical students. Therefore, on-site framework conditions always tailor the offers to the target group. The preferred forest landscapes of the user group surveyed are untouched. Forests, mixed forests, open and light forests, and well-groomed forests. This also corresponds to criteria regarding forest aesthetics. All of these are not very popularly managed forests. This underscores the tension between forest owners and visitors; it was also a hot topic in the expert interviews. Forest owners must live from forestry and are dependent on forest management. Forest visitors want to experience untouched nature that is well-cared for. These different interests will work together in the future. The majority of the students surveyed think that

- Awareness-raising about the health effects of the forest would be important in the future.
- The topic of the health effects of the forest gain becomes important.
- The forest plays a major role in health prevention.
- The forest supports medical treatments.
- The forest in the vicinity of a clinic affects the patient's wellbeing.
- The forest owners are interesting cooperation partners for the medical facility.

This feedback is encouraging and shows that the positive effects of the forest are valued. There is always a noticeable openness to forests and health for medical students. However, the target group surveyed needed more information about the health effects of the forest. At the open, feedback regarding the wishes of the forest was communicated, and more information and communication about it were needed. More information and communication are also issues in forestry itself. Concrete offers are few for the subjects of the written survey known, and only a few can imagine paying for such offers. In addition, the respondents would like a more restricted approach to forestry management and greater forest opening. This topic is reflected in the expert interviews from another perspective. The forest is called something that is taken for granted. This reduces the willingness to pay for products. Therefore, it is used in the forest and health products and needs services that have an additional benefit beyond the recreational function.

## 8. Conclusion

Forestry is characterized by a strong traditional way of thinking shaped over the centuries. Forestry products are primarily timber production and hunting. The understanding of forestry for the desires of forest visitors is only available to a limited extent. New things are often met with great reservation. Innovation and development are currently playing a minor role. There are, of course, innovations, for example, about the efficiency of wood production, but hardly in the area of product innovations.

### 8.1. The forest strategy

It requires a definition of packages of measures with clear responsibilities and binding time frames. A possible model would be, e.g., strategy “leisure and recreation in the forest”. A second possibility would be that the stakeholders involved in the forest strategy take responsibility for implementing packages of measures in the form of binding self-commitment takeovers. Another important step would also be the development of meaningful indicators, e.g., awareness or competence expansion of the health effects of the forest in the population map, for example, through representative surveys, a number of state-recognized training and further education measures, health professions in forest and health and others.

Forest and health brand requires developing and establishing a “Forest and Health” brand in Saudi Arabia. There is also one associated with this professional communication strategy for forestry (internal), for the Arabian population (to the outside), and for possible cooperation partners, e.g., for the health sector, tourism, or company. This also requires clear process ownership. Potential candidates include the Chamber of Agriculture, the Arabian Forstverein, and the Federal Ministry for Tourism and Sustainability.

### 8.2. Facts and figures

Another important measure is the establishment of exemplary visitor monitoring in urban areas or strong tourist regions. The aim is to assess the motives, behavior, and activities of the forest visitors, e.g., their impact on the ecosystem and forestry use, to assess more precisely. Over and beyond, visitor monitoring represents an argumentation aid about politics and society to better represent one's interests. It also serves as a basis for decision-making for effective visitor management.

### 8.3. Innovation and development

Forestry establishes or participates in innovation clusters in the regions, aiming to develop innovative products and economically relevant services jointly. It is binding cooperation at the regional level between different research areas and partners from industry and science.

### 8.4. Digitization

Development of a digital platform on the subject of forest and health in which people find out about products and services throughout Saudi Arabia and information about the health effects of the forest. At the same time, she could serve as an exchange platform between forestry and forest visitors. In addition, apps on the subject of forests and health can be linked to concrete instructions developed.

### 8.5. Education

In training and further education for forest experts and owners, investments should also be made in health professions concerning forests and health. As a first step, the goal could be to raise awareness and develop communication skills for this topic with these target groups. In a second step, people need to be empowered for this field of work, for example, within the framework of advanced courses for health professions or those trained in forestry.

Integrative forest management is built up in urban areas or tourist regions. The participation of stakeholders in decision-making and development processes is in focus. This can already be done when creating forest development plans or joint projects on forest and health. In addition, professional communication and public relations are required. Furthermore, it requires mediation skills to balance economic, ecological, and social interests and solve conflicts. It would be important for Arabian society to develop incentive systems for creating forest owners to address the topic of “health effects of the forest”, which is easier to establish in forestry. The theoretical processing of the topic and the ten expert interviews provide a good insight into and overview of the current challenges in forestry on the topic of the health effects of the forest. In addition, possible connections within the framework of Green Care Forest between the health sector and forestry are presented, and ideas for strategic and operational measures are derived.

The validity of the quoted scientific studies on “health effects of the forest” is not always comprehensively scientifically proven. Further studies in a randomized, controlled form are planned for future topics. The quantitative research part is due to the small number of participants that need to be regarded as representative. Still, it only gives an impulse about the user behavior of young adults undergoing medical training. The selection of this group was interesting because future cooperation with partners in forestry could be possible. Making statements about user behavior would require a much larger sample representing the Arabian population.

In the proposed research, other interesting topics arose that are essential for forestry research and should be further explored. These findings are the limitations of the current work and should be further investigated in the future and deepened in the context of further research. A few research areas could be as

- innovative product development for the health effects of the forest in the context of digitization, e.g., in the form of apps.
- establishment of a “Forest and Health” brand in Saudi Arabia.
- integrative forest management in the urban environment.

## Data availability statement

The original contributions presented in the study are included in the article/Supplementary material, further inquiries can be directed to the corresponding author.

## Author contributions

AA: Formal analysis, Funding acquisition, Investigation, Methodology, Writing—original draft, Writing—review and editing, Conceptualization.
