# Supplementary material for: Impact of forestry on environment and human health: an evidence-based investigation
Source: Front Public Health. 2023 Sep 1;11:1260519. doi: 10.3389/fpubh.2023.1260519 (PMC10513052; doi:10.3389/fpubh.2023.1260519)
Supplement: Supplementary file 1 [file Data_Sheet_1.pdf]

frontiers

Appendix

Appendix 1: Interview guidelines for the experts

Expert interview

1) Please introduce yourself and outline your work or expertise.
2) Which developments and challenges are currently or in the in the next 10 years in Saudi Arabian forestry?
3) What developments/trends do you observe in relation to the health effect of the woods
   a. in forestry?
   b. in society?
4) Do you see potential for the Saudi Arabian in the health effects of the forest? How could this be specifically promoted?
5) In your opinion, how do the users of the forest affect their health? after
   a. on the forest ecosystem?
   b. on the economic use of the forest?
   c. Who do you think are the main user groups currently/in the future?
6) Which strategic and practical measures would be useful from your point of view, thus a balance between
   - the users of the health effects of the forest
   - the forest ecosystem
   - and the economic use of the forest is to create?
7) Is there anything else you would like to add that we haven't talked about in the interview have spoken?

Appendix 2: Written Survey

The survey is written to get more information about the effect of forest. The focus is on health effects of the forest, which is why the wishes/needs in the survey be queried from the point of view of the users of the forest. Participation in the survey is voluntary and anonymous, all results are treated confidentially and can not be tracked. Thank you for your support!

1. Gender: o female o male
2. Year of birth: _______________
3. The nearest forest is about ____________________ km away from where I live
4. When you think about your last stay in a forest - what comes to mind spontaneously come to mind?

---

5. How often do you do the following activities?

	Daily	Times/Week	Time/Month	Rarely	Never
I'm in the forest					
I walk in the woods one or two walk for hours.					
I walk a half in the woods hike for a day or more.					

I meditate in the forest.					
I do sports in the forest.					
I work in the forest.					
I relax in the forest.					
Other activities:					

6. How much do you agree with the following statements?
   (1 = I strongly agree; 5 = I completely disagree; N/A = No answer)

	1	2	3	4	5	N/A
A stay in the forest is important for my general well-being.						
Staying in the woods helps me when I'm not doing well mentally.						
Staying in the forest helps me when I have physical pain.						
Staying in the forest helps me to relieve stress.						
Staying in the woods helps me find new ones to develop perspectives.						
Staying in the forest promotes my social activities.						
The forest is important to me recreation room.						
Staying in the forest is important for my health.						

7. In the forest are important to me….
   (1 = very important; 5 = not important at all; N/A = not specified)

	1	2	3	4	5	N/A
Quiet						
Cleanliness						
Rest stops						
Walkable network of paths						
Orientation options						
Biological diversity						
Animals						
Plants						
Mushrooms						
Health-promoting offers						
Sports facilities (mountain biking, jogging, hike …)						
Knowledge transfer						
Barrier-free paths						
Toilet facilities						

8. Please rank the following forest landscapes according to your personal preference (from 1 = I prefer to be here to 8 = I do not like to be here at all)

In pristine forests	
In near-natural managed forests	
In heavily used forests	
In open sparse forests	
In dense forests	
In mixed forests	
In monoculture forests	
In manicured forests	

9. Health effects of the forest (1 = very much; 5 = not at all; n/a = no answer)

- Health effects include health prevention such as burnout prevention, strengthening of the immune and cardiovascular systems, and on the other treatments such as therapy support for mental or chronic diseases understood.

	1	2	3	4	5	N/A
I am well informed on this subject.						
I would like to get involved more intensively in the future deal with this topic.						
I would be open to offers as well to pay.						
I'm already paying for preventive health measures in the forest.						
Raising awareness about the health effect forest would be in the society important.						
The topic wins in the future for the Saudi Arabian society in importance.						
The Saudi Arabian forest owners make sufficient offers in this regard available.						

10. Forest meets medicine (1 = very much; 5 = not at all; n/a = no answer)

	1	2	3	4	5	N/A
I know of scientific studies on the health effects of the forest.						
I think the forest plays one important role in preventive health care the population.						
I think the health effects of the forest can support medicinal treatments.						
I think the forest around a clinic encourages that well-being of the patients.						
I think forest owners would be interesting Cooperation partners for medical facilities (e.g. rehabilitation, psychiatry, houses).						

11. Three wishes to the Saudi Arabian forest owners on the subject health effects of forests….

____

____

____
